# Disentangling Mechanisms Behind Chronic Lethality through Toxicokinetic–Toxicodynamic Modeling

**DOI:** 10.1002/etc.5027

**Published:** 2021-05-04

**Authors:** André Gergs, Jutta Hager, Eric Bruns, Thomas G. Preuss

**Affiliations:** ^1^ Environmental Safety, Bayer CropScience Monheim Germany

**Keywords:** General unified threshold model of survival, Dynamic energy budget, Insecticides, Invertebrates, *Chironomus riparius*, Feeding inhibition

## Abstract

Ecotoxicological profiles of the 3 insecticides imidacloprid, thiacloprid, and flupyradifurone in terms of acute and chronic effects were analyzed in *Chironomus riparius*. Toxicokinetic–toxicodynamic modeling revealed that chironomids would die from starvation as a result of prolonged feeding inhibition under chronic exposures. The starvation effect is an indirect cause for mortality, which, for the neonicotinoids, adds to the direct/acute mortality, although the results suggests that this additional effect is not relevant for flupyradifurone. *Environ Toxicol Chem* 2021;40:1706–1712. © 2021 Bayer Inc. *Environmental Toxicology and Chemistry* published by Wiley Periodicals LLC on behalf of SETAC.

## INTRODUCTION

The environmental risk assessment of pesticides is based on acute and chronic toxicity tests with various biological species. Within the aquatic assessments, acute tests are carried out for short periods, usually a few days, with a focus on lethality. The focus of chronic testing is on sublethal endpoints such as organismal growth and reproduction, but also lethality is frequently considered as a chronic endpoint. Moreover, chronic tests are conducted for longer periods, usually several weeks. The effective concentrations derived from summary statistics such as the no‐observed‐effect concentrations or the *x*% effect concentration are frequently lower in chronic tests compared to acute time frames (i.e., the apparent sensitivity may increase over time; see Van den Brink et al. 2016). Moreover, effect concentrations can be lower for sublethal endpoints compared to lethal ones. This should particularly be true when examining individual organisms and taking the temporal dynamics of chemical uptake into account because, by definition, sublethal endpoints occur before the organism dies.

Test designs mostly differ among acute and chronic tests with the same test species (e.g., in that the provision of food is more common in chronic setups). Also, the exposure situation may differ between acute and chronic tests. In tests with the nonbiting midge *Chironomus riparius*, water‐sediment systems are used in the chronic trials (Organisation for Economic Co‐operation and Development 2004), whereas a water‐only exposure is applied in the acute counterpart (Organisation for Economic Co‐operation and Development 2011). The water‐only exposure is supposed to limit declines of exposure concentrations within the acute time frames. In contrast, the use of sediments in chronic spiked water setups usually results in declining water concentrations through adsorption to particles if static exposure is applied.

Toxicokinetic–toxicodynamic (TK‐TD) models explicitly integrate exposure and effects in a process‐based manner. As such, these models allow prediction of toxicity for various exposure situations and thus help to explore potential differences among test designs. They explicitly simulate processes leading to an effect such as bioaccumulation and/or damage dynamics and thereby allow making predictions for the effect of exposure time on apparent toxicity (Jager et al. 2006).

As for survival, different TK‐TD model formulations have largely been integrated within the general unified threshold model for survival (GUTS; Jager et al. 2011). The GUTS framework has been applied to predict survival for time‐variable exposure situations (Ashauer et al. 2013; Nyman et al. 2013), explore toxicodynamic recovery times (Ashauer et al. 2013), and investigate differences in sensitivity across life stages and species (Kulkarni et al. 2013; Gergs et al. 2015, 2016). Because GUTS theoretically allows prediction of survival probabilities at any given point in time, validation attempts have been made to extrapolate toxicity at chronic timescales based on acute test results (Focks et al. 2018).

Although within the GUTS framework a constant physiological state of the organism is generally assumed, bioenergetic models explicitly address organismal growth, development, and reproduction, which are relevant in chronic timescales. Dynamic energy budget (DEB) models (Kooijman 1986, 2010) are particularly suitable to simulate organism performance throughout the organism's entire life span. They consider temporal dynamics of energy acquisition from the environment and energy allocation to bodily functions and are, thus, applicable to variable environmental conditions. In the context of ecotoxicology, DEB models make use of physiological modes of actions (e.g., reduced feeding or increased maintenance costs) to simulate changes in life‐history processes, such as growth, maturation, and reproduction, on chemical exposure (Jager and Zimmer 2012). Under prolonged feeding inhibition, which is effectively similar to the absence of food, bioenergetics might be affected to an extent that the organism ultimately dies from starvation (Gergs and Jager 2013).

In the present study, we explored acute and chronic effects caused by the insecticides imidacloprid, thiacloprid, and flupyradifurone on *C. riparius*. Our aim was to explore underlying mechanisms in chronic toxicity of those compounds by TK‐TD modeling. In a first step, we analyzed acute and chronic mortality using the GUTS framework. Second, we applied a DEB model to describe the developmental delay observed for chronic exposures and analyzed the potential of chemically induced starvation.

## MATERIAL AND METHODS

### Experimental data

Acute toxicity and chronic toxicity of imidacloprid, thiacloprid, and flupyradifurone were separately tested in *C. riparius*. First‐instar larvae, 1 to 3 d of age, were used to initiate the experiments. Prior to experiments, chironomid egg clutches were collected from a laboratory breeding, transferred in vessels containing artificial freshwater (see Organisation for Economic Co‐operation and Development 2011), and acclimatized to test conditions. During acclimatization, larvae were provided with small amounts of a commercial ornamental fish food (Tetra Phyll®).

Acute toxicity tests were carried out following the Organisation for Economic Co‐operation and Development guideline 235 (2011). Briefly, in the standard tests, chironomid larvae were exposed in groups of 5 to different concentrations of the test item for 48 h in a static manner. The number of immobilized larvae in the control and treatments was recorded after 24 h and at test termination. Immobility was assessed via mechanical stimulation of the organisms. In addition to the standard tests, toxicity was assessed in pulse exposure studies generally following the same experimental setup. However, deviations from the Organisation for Economic Co‐operation and Development guideline 235 were as follows: pulse exposure studies encompassed 3 dose rates of the test item and a control, replicated 2 times. The larvae were exposed to 2 pulses, on days 0 and 1, respectively. In 2 different scenarios, pulse durations of 4 and 8 h were employed. Peak exposure was terminated by transferring the larvae to untreated medium. The pulse durations were chosen to account for the relatively fast toxicokinetics/damage dynamics of the tested chemicals. The mobility of the larvae was determined several times by visual observation following a stimulus to trigger movement during the 48‐h test period. The exposure concentrations for the 3 different chemicals and the different scenarios as applied in the toxicity trials are given in Supplemental Data, S2.

Chronic tests followed Organisation for Economic Co‐operation and Development guideline 219 (2004) to assess the influence of the test items on emergence and development of *C. riparius* in 28‐d bioassays in static water‐sediment systems (spiked water). Tests were initiated with first‐instar larvae, and Tetra Phyll was provided as a resource at least 3 times per week. The time point of adult emergence and the number of emerged midges were recorded daily during the period of emergence. Because only fully emerged adults were relevant for the endpoints of the present study, larvae which did not yet mature were not accounted for in emergence rates and development times. In cases where no emergence was recorded, the developmental rate was assumed to be 0. Lethal chronic effects were inferred from 1 minus the fraction of emerged chironomids compared to the initial number; that is, the emergence rate was used as a proxy for survival. Water concentrations were quantified 3 times during the chronic tests (initially, on day 7, and at termination) by high performance liquid chromatography (HPLC)‐ultraviolet and HPLC‐tandem mass spectrometry. Measured concentrations in overlying water at test termination were on average 10.7% (thiacloprid), 24.3% (imidacloprid), and 49.4% (flupyradifurone) of nominal. We, thus, used measured concentrations in our model analysis (see section, *Effect models*). Further details are laid out in the original study reports, which are available on request. For a list of studies and contact details, see Supplemental Data, S1.

### Effect models

The TK‐TD modeling of survival is, in a first attempt, based on the GUTS framework. We applied the reduced version of the GUTS model (GUTS‐RED), which makes use of a scaled damage state. The associated dominant rate constant represents either toxicokinetics or toxicodynamics depending on the rate‐limiting process. Furthermore, the 2 toxicodynamic assumptions of stochastic death and individual tolerance were employed, hereafter referred to as GUTS‐RED‐stochastic death and GUTS‐RED‐individual tolerance, respectively. For further details see, for example, the GUTS introduction by Jager and Ashauer (2018). The GUTS‐RED models were calibrated based on the acute data as outlined in the previous section. The immobility endpoint was used as a proxy for mortality. However, because organismal recovery from immobility was possible in the pulse exposure studies and GUTS does not allow for a decreasing number of affected individuals over time, the acute data were censored prior to calibration: for each time point, the maximum immobility since the start of the experiment was calculated (i.e., if decreasing immobility at time *t* was observed, the number of affected individuals at *t* – 1 was used as the model input). Calibration was performed in the open‐source statistical software R, Ver 3.0.0 (R Development Core Team 2018), applying the GUTS implementation in the R package “morse,” Ver 3.2.0 (Baudrot et al. 2019), which in turn applies the open‐source Gibbs‐sampling software jags, Ver 4.3.0 (Plummer 2017). Model performance of the calibration step was assessed based on the criteria recommended by the European Food Safety Authority (EFSA Panel on Plant Protection Products and Their Residues 2018), that is, the posterior predictive check (PPC), the normalized root mean square error (NRMSE), and the survival probability prediction error (SPPE), which focus on different aspects of the model performance. Calibrated GUTS‐RED models were subsequently executed to predict lethal effects in the chronic trials, taking the time course of the static exposure into account. The decline in chemical concentration in the static sediment‐water setup was represented by single‐first‐order kinetics and a chemical‐specific rate constant, *k*, estimated from the measured water concentrations. For model testing, GUTS‐RED predictions (mean and 95% confidence intervals) were compared to the concentration response of the lethal effects observed at termination of the chronic tests.

In a second step, we analyzed chronic toxicity using an existing DEB model for *C. riparius*. The model code, parameters, and underlying data are freely available from the add‐my‐pet collection (Marques et al. 2018). The current model version (Augustine and Gergs 2019) was changed to allow for increasing larval development times with decreasing food assimilation: in the modified model, pupation is initiated at a certain threshold of the structural length rather than at a threshold of the reproduction buffer density (for reasoning and implications, see section *Results and Discussion*). The DEB model for *C. riparius* was extended by a TK‐TD module, where the stress level on the DEB parameter(s), corresponding to a physiological mode of action, is based on the level of the scaled damage, the latter being the same as in GUTS‐RED‐stochastic death (i.e., the same parameter value is used). For details on the physiological mode of actions see, for example, Jager and Zimmer (2012). The reasoning using the damage dynamics as derived from the stochastic death toxicodynamic assumption is that both DEB‐TK‐TD and GUTS‐RED‐stochastic death make use of a threshold for the onset of the effects that is the same for all individuals, and the stochastic death formulation is originally derived from DEB survival modeling.

The DEB‐TK‐TD model was used to analyze the results from the chronic experiments, with respect to the development rate (the inverse time to emergence) and starvation‐induced mortality. For model calibration, first the feeding level (scaled functional response in the DEB model) was adjusted to meet the development rate in the controls. For each chemical, only 2 parameters, the threshold for sublethal effects and the tolerance concentration, were estimated from the chronic concentration responses. The standard DEB parameters remained untouched, and the dominant rate, *k*_d_, was fixed to the values derived from the GUTS parametrization; for a discussion, see the following section. Model calibration is done using DEBtool (Kooijman et al. 2017), following the methodology outlined in Lika et al. (2011). The same exposure scenario as in the GUTS prediction was used in the DEB‐TK‐TD model calibration. In accordance with the reported experimental results, the modeled development rate was assumed to be zero if the time to adult emergence may have exceeded the duration of the chronic test or 100% mortality occurred.

## RESULTS AND DISCUSSION

The GUTS‐RED models were calibrated based on data derived from standard acute toxicity studies and acute studies employing time‐variable exposure conditions. An overview of the resulting parameter values obtained for the 3 chemical compounds flupyradifurone, imidacloprid, and thiacloprid is provided in Table 1. A visual comparison of model fits and data as well as information on the confidence limits of the parameter estimates are available in Supplemental Data S1. For all 3 compounds, the standard acute data based on constant exposures and the high immobility in the pulse exposure test were well described by the GUTS‐RED models. For pulse exposures and intermediate effects, there is a tendency for underestimation of immobility. This might to some extent be a result of the data censoring (using the maximum immobility over time) prior to GUTS calibration: because organismal recovery was not considered in the GUTS input, the observed effects in the pulse exposure studies were higher compared to the standard tests, where recovery was not possible because of the constant exposure situation. Because GUTS‐RED models were fit to both data types simultaneously, the bias due to data censoring is leveled out, hence the lower effect prediction compared to data in the concerned treatments. However, overall, GUTS‐RED models fit the data for the 3 chemicals well, as indicated by the model performance criteria: in all cases the values for the PPC and the NRMSE were >91 and <45%, respectively. Some larger differences (>20% deviation) between GUTS‐RED fit and data at the end of the test were mainly visible for intermediate effect levels in the pulse exposure studies, as indicated by the SPPE (for details, see Supplemental Data, S1).

**Table 1:** Median general unified threshold model for survival reduced parameter estimates for the 2 toxicodynamic assumptions stochastic death and individual tolerance based on acute toxicity data from *Chironomus riparius* testing^a^

		Flupyradifurone		Imidacloprid		Thiacloprid	
Parameter	Unit	SD	IT	SD	IT	SD	IT
Dominant rate (*k*_d_)	h^–1^	2.59	0.000764	0.0914	0.0635	0.0881	0.00550
Background hazard rate (h_b_)	h^–1^	0.00133	0.00113	0.00469	0.00431	0.00281	0.00211
Threshold (z)	mg L^–1^	0.0284		0.0502		0.0179	
Killing rate (k_k_)	L mg^–1^ h^–1^	0.361		64.8		35.6	
Threshold median (α)	mg L^–1^		0.00189		0.0472		0.00314
Spread of threshold (β)	—		3.22		13.5		7.26

^a^ All input data for parameter estimation are given in Supplemental Data, S1 (see also for visual assessments of the model fits, confidence limits, and model evaluation).

SD = stochastic death; IT = individual tolerance.

**Figure 1:**
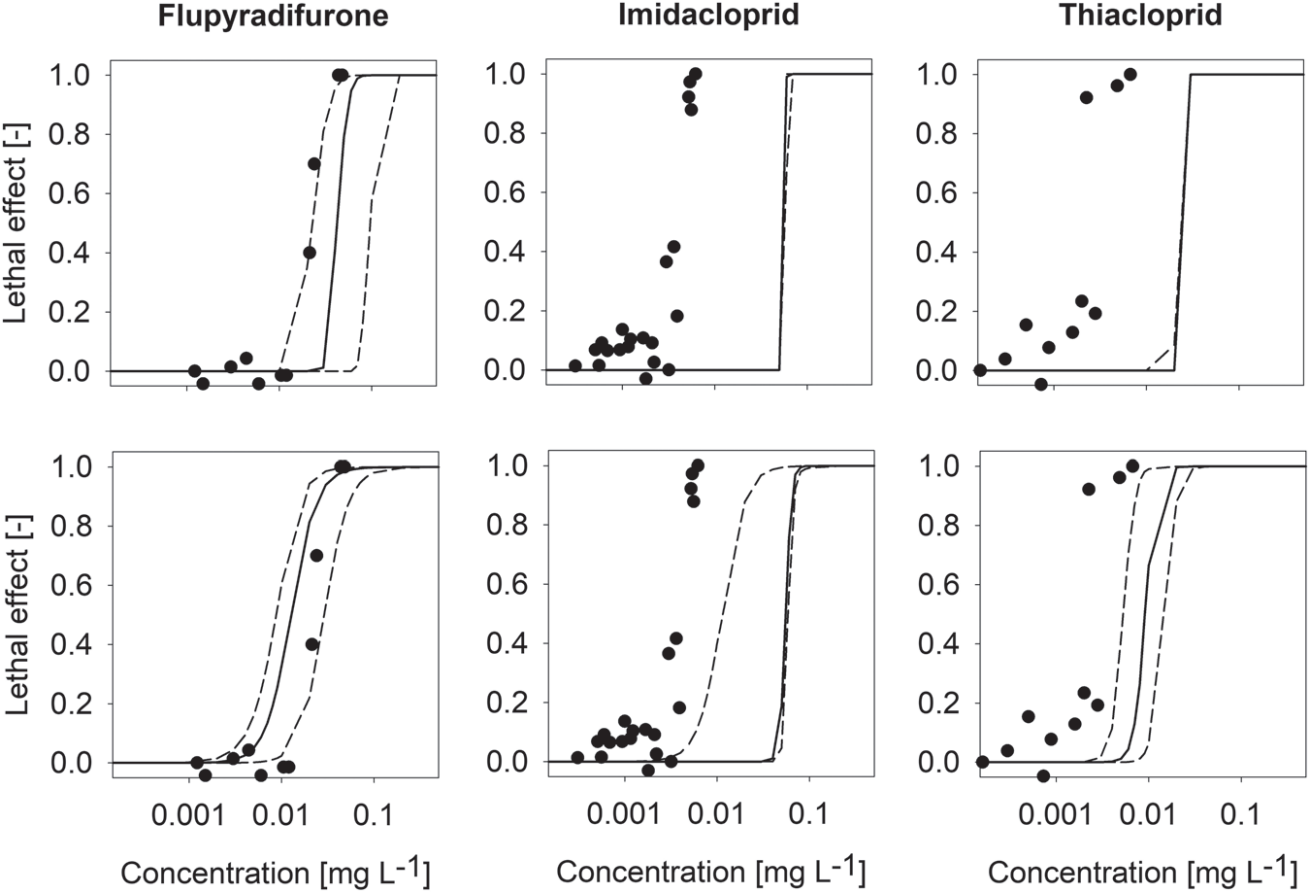
Chronic 28‐d lethality as a function of exposure concentration. Dots are measured data from water‐sediment toxicity tests with *Chironomus riparius*. Solid and dashed lines represent the median and 95% confidence limits of predictions by the general unified threshold model for survival reduced (GUTS‐RED) assuming stochastic death (upper panel) and the GUTS‐RED assuming individual tolerance (lower panel). The GUTS‐RED models had been parameterized based on acute toxicity data (see Supplemental Data, S1). The *x*‐axes refer to initial measured concentrations, and each dot represents the mean of 4 to 8 replicates with 20 chironomids each.

The parameterized GUTS‐RED models were subsequently applied to make independent predictions for survival as observed in the standard chronic tests (Figure 1). Therefore, the longer time frame of 28 d and the decline in exposure concentration were explicitly accounted for. The chemical‐specific values for the dissipation rate used in the model are listed in Supplemental Data, Table S1‐13. For flupyradifurone, the chronic concentration response was reasonably well predicted by the GUTS‐RED models. However, differences exist among the 2 different toxicodynamic assumptions: while in GUTS‐RED‐stochastic death, the predicted effect concentrations were somewhat higher than observed, the effects were slightly overestimated by the individual tolerance counterpart (Figure 1), but all of the data points on the slope of the concentration response were within the 95% credibility interval of the GUTS‐RED‐individual tolerance prediction. This is in line with theory because the 2 toxicodynamic assumptions, stochastic death and individual tolerance, can be seen as 2 extreme representations of the death mechanism, with the real system behavior somewhere in between. In contrast, in the case of imidacloprid and thiacloprid, the concentration response for chronic mortality was not well predicted; the observed effect concentrations were in the order of 1 order of magnitude lower compared to the simulated ones (Figure 1).

A similar mismatch in GUTS performance between acute and chronic survival has been reported by Focks and coworkers (2018) for the case of the mayfly *Cloeon dipterum* and 3 different neonicotinoids: imidacloprid, thiacloprid, and thiamethoxam. The authors separately calibrated GUTS based on acute and chronic data sets. The obtained parameter sets were remarkably different between the 2 calibration attempts. For instance, the GUTS threshold parameters derived from the calibration with acute data were 1 order of magnitude higher compared to the ones based on chronic data; this is in line with our observation for the cases of imidacloprid and thiacloprid of chronic effect concentrations being lower than predicted by GUTS based on acute data. One reason for the failing GUTS validation with chronic data in the case of the 2 neonicotinoids in the present study might be that the acute data were not sufficiently informative; that is, the data do, for instance, not allow one to estimate the “real” effect threshold, to make adequate predictions for chronic timescales. Consequently, Focks et al. (2018) suggested that both data sets, acute and chronic, could be used for the calibration step. However, our hypothesis is a different one, as follows.

**Figure 2:**
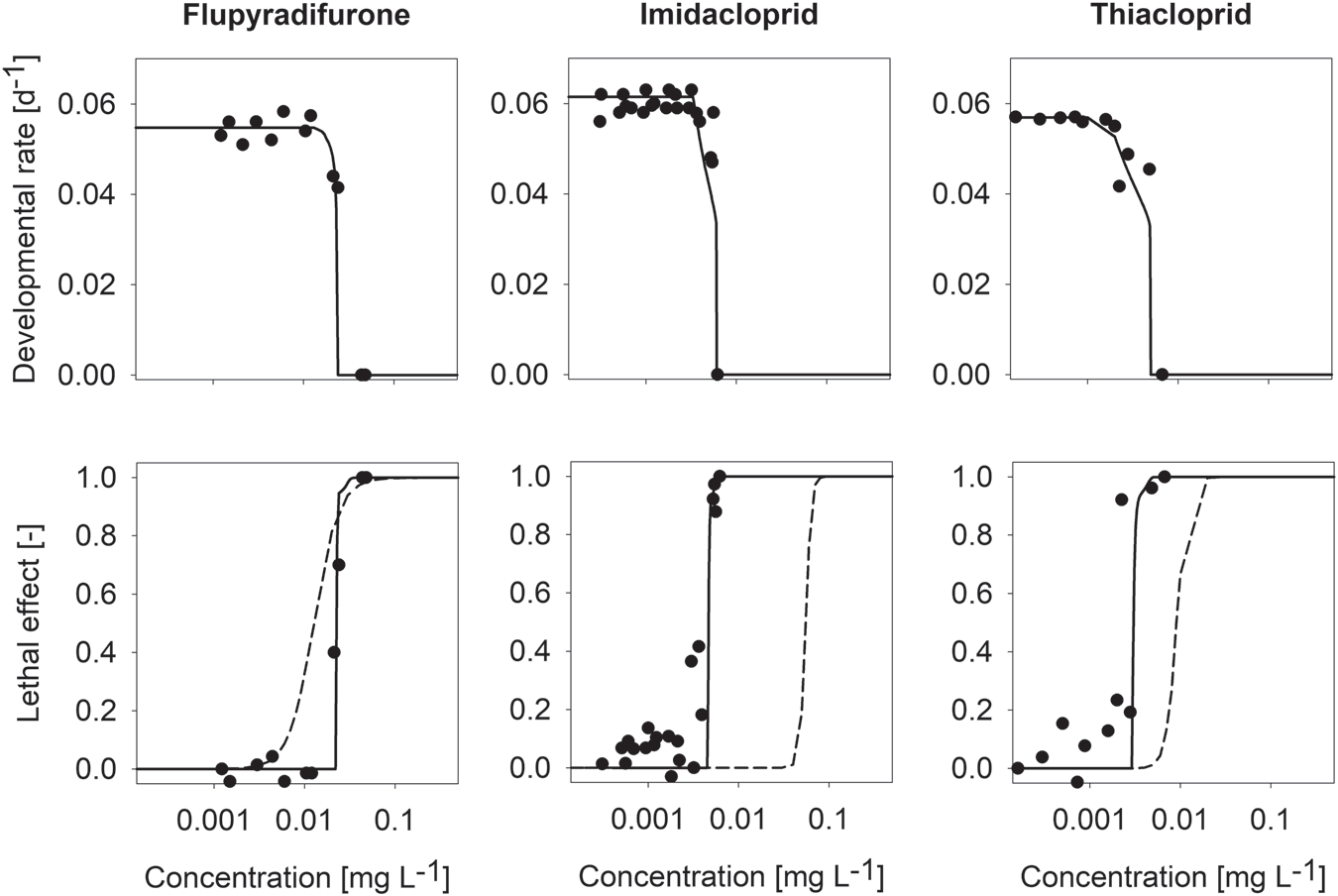
Developmental rate and lethal effect as functions of concentration for the 3 tested insecticides. Dots are measured data from chronic 28‐d toxicity tests with *Chironomus riparius*. Solid lines represent the calibrated dynamic energy budget models for the developmental rate and starvation induced mortality on simulated feeding inhibition. In the lower panel, the median prediction of general unified threshold model for survival reduced assuming individual tolerance (Figure 1) serves as a reference (dashed line). The *x*‐axes refer to initial measured concentrations, and each dot represents the mean of 4 to 8 replicates with 20 chironomids each.

We assumed that the mismatch between the chronic survival data and the GUTS prediction for neonicotinoids is due to additional effects that are not covered by the survival model based on acute information. For instance, feeding inhibition on exposure to neonicotinoids has been observed in several aquatic species (Alexander et al. 2007; Pestana et al. 2009; Agatz et al. 2013; Nymann et al. 2013; Arican et al. 2017). Feeding impairment may result in reduced assimilation of energy and subsequently in chronic effects such as reduced growth and reproduction (see Jager et al. 2006). Similarly, in our studies with imidacloprid, thiacloprid, and flupyradifurone, developmental delays were observed in *C. riparius* (Figure 2). Our hypothesis is, thus, that in the chronic experiments prolonged feeding inhibition led to indirect effects on survival as a result of starvation.

We tested this hypothesis by applying a DEB model parametrized for *C. riparius*. The model describes the growth of chironomid larvae under different food conditions and the starvation resistance of first‐instar larvae well (Augustine and Gergs 2019). It is important to note that in our model application starvation was the only cause for mortality and only a single mechanism was used to describe both the sublethal and the lethal effects. The DEB‐TK‐TD model reproduces the development and survival data from the chronic toxicity trials with imidacloprid, thiacloprid, and flupyradifurone well (Figure 2). The resulting DEB‐TK‐TD model parameter estimates and the model evaluation statistics are listed in Supplemental Data, Table S1‐13. The difference between GUTS prediction and actual survival in the neonicotinoid‐exposed chironomids can be explained by the applied physiological mode of action of feeding inhibition in the DEB model, which is in line with our hypothesis. To understand the implications, it is important to realize that the GUTS prediction represents the direct (acute) toxicity, whereas the starvation effect mimicked by the DEB model is an indirect result of the sublethal effects under prolonged exposure. This difference is, however, a minor one in the case of flupyradifurone (Figure 1); that is, the additional effect is not relevant for this compound.

There are a few points in the model assumptions that need some attention. First, we used the same values for the dominant rate constant *k*_d_ in GUTS as well as in the toxicity module of the DEB model, which implies that the same damage mechanism is responsible for both immobility and sublethal effects. This assumption was made to account for the lack of temporal effect information from the chronic toxicity tests with *C. riparius* where apical endpoints are only quantified at the end of the 28‐d period. Moreover, the concentration responses of the tested molecules are steep, making it difficult to assess the full range of developmental effects empirically. This might have had an impact on the estimation of the toxicodynamic parameters in the DEB model but will not affect our overall conclusions in terms of underlying mechanisms.

Both neonicotinoid insecticides and the butenolide flupyradifurone interact with insect nicotinic acetylcholine receptors (nAChRs) in an agonistic manner. In contrast to the neonicotinoids, flupyradifurone contains the distinctive butenolide pharmacophore system and has the unique *N*‐2,2‐difluorinated ethyl side chain (Nauen et al. 2015). Binding of nAChR agonists to the neurotransmitter‐gated cation channels induces depolarizing ion currents and subsequent excitation of the nerve cells. The compounds cannot be inactivated by acetylcholinesterase, thus binding results in a disorder of the nervous system and subsequently in immobility symptoms and death. We used the immobility endpoint as a proxy for survival in our GUTS analysis but note that acute mortality may occur at higher effect concentrations or that the mortality effect is lower compared to immobility at a given concentration and point in time (see Macaulay et al. 2020; see also reports for pulse exposure studies as listed in the Supplemental Data). Acute mortality will, thus, not be a good predictor for chronic effects, also in the case of flupyradifurone.

We assumed that feeding inhibition is the primary physiological mechanism behind chronic effects, based on previous reports of feeding impairment caused by neonicotinoids (Alexander et al. 2007; Pestana et al. 2009; Agatz et al. 2013; Nymann et al. 2013; Arican et al. 2017). Pestana et al. (2009) also observed increased respiration rates with increasing imidacloprid concentrations in *C. riparius*, but the opposite trend was found for the caddisfly *Sericostoma vittatum*. In the DEB context, increased respiration can be associated with increased maintenance costs, which in turn will lead to reduced growth and development. In the applied DEB starvation module, the hazard increases if maintenance costs cannot be paid, and the organism starts to shrink in terms of somatic structure, irrespective of the type of stress, be it reduced feeding or increased maintenance costs. Our overall conclusion of an additional mechanism resulting in mortality in chronic tests with neonicotinoids is, thus, not affected by the choice of the physiological mode of action in the DEB toxicity module. Future trials may directly quantify feeding inhibition to predict chronic effects, which would allow an independent model validation based on chronic test results.

The originally published DEB model for *C. riparius* (Augustine and Gergs 2019) made use of the reproduction buffer density as a trigger of pupation and subsequently emergence of the adult midges. As a consequence of this model, chironomids would obtain smaller body sizes at pupation and produce smaller egg clutches at reduced food levels compared to ad libitum but maintain the same development times. The opposite was, however, observed by Péry et al. (2002): development time remarkably increased with food deprivation, whereas the maximum length did not significantly differ across the different food levels. In our current modification of the DEB model, we used structural length as a trigger for the onset of pupation (rather than reproduction buffer density), which allows for longer development times as food availability is reduced; but reproduction is unaffected at low food densities, which is also not realistic. We, however, ignored this part of the energy budget because the focus in our study was on development times and survival. Largely ignoring the part of the energy budget related to reproduction would potentially affect starvation resistance because in the DEB context maintenance costs can principally be paid from the reproduction buffer. This mechanism is, however, unlikely to affect our results because in the simulated experiments starvation already strikes within the first days of exposure, when the larvae are small and the contribution of the reproduction buffer is negligible. Nevertheless, further research is needed to verify the generality of the trade‐offs under food limitation in insects as observed by Péry et al. (2002) and to develop DEB models that adequately cover the complete pattern of life‐history traits in those species.

In summary, we found that for flupyradifurone the chronic mortality endpoint was well predicted by the GUTS‐RED models as parametrized based on acute toxicity data. In contrast, for imidacloprid and thiacloprid, chronic effect concentrations were approximately 1 order of magnitude lower compared to the mortality as predicted from the acute experiments. The assumption of feeding inhibition in the DEB model described the delayed emergence of chironomids in the chronic trials well. Moreover, the DEB model analyses revealed that under prolonged exposure to flupyradifurone or neonicotinoids the chironomids would eventually die from starvation because of reduced food intake. The starvation effect is a second, indirect cause for mortality, which adds to the direct (acute) effect on mobility on chemical exposure. This observation can explain the mismatch between chronic mortality and GUTS prediction in imidacloprid and thiacloprid. Contrastingly, for flupyradifurone, the effect concentrations for indirect mortality and the acute effect are within the same order of magnitude; and thus, the chronic mortality was well predicted by GUTS (i.e., the additional chronic effect is not relevant for flupyradifurone). This demonstrates that the butenolid flupyradifurone has an ecotoxicological profile different from the neonicotinoids.

The presented case study illustrates that in chronic assessments lethal effects may be as relevant as the sublethal endpoints. The choice of the effect model to adequately address the effects should, thus, be based on the actual mechanism leading to an effect. In general, GUTS is considered the appropriate TK‐TD model for addressing lethal effects in risk‐assessment refinements. If GUTS validation with chronic data fails, this might in the first place be a consequence of inappropriate calibration data. Focks et al. (2018) concluded that, for substances like the neonicotinoids, GUTS calibration should, thus, be based on acute and chronic data together to achieve a good model fit. In general, having more information available for calibration will likely increase model performance. However, in cases where additional effects come into play, for example, in chronic timescales, different model approaches might be needed to adequately cover the underlying mechanisms, such as a DEB model covering death on starvation under prolonged feeding inhibition.

## Supplemental Data

The Supplemental Data are available on the Wiley Online Library at https://doi.org/10.1002/etc.5027.

## Disclaimer

The authors are employed by Bayer. Imidacloprid, thiacloprid, and flupyradifurone are active ingredients used in Bayer products.

## Supporting information

This article includes online‐only Supplemental Data.

Supporting information.

Supporting information.

## Data Availability

Underlying data and full study reports can be requested by sending an email to cropscience-transparency@bayer.com. Data, associated metadata, and calculation tools are also available from the corresponding author (andre.gergs@bayer.com).
