# Supplementary material for: Disentangling Mechanisms Behind Chronic Lethality through Toxicokinetic–Toxicodynamic Modeling
Source: Environ Toxicol Chem. 2021 May 4;40(6):1706–12. doi: 10.1002/etc.5027 (PMC8252366; doi:10.1002/etc.5027)
Supplement: Supplementary file 1 — Supporting information. [file ETC-40-1706-s002.docx]

Supplementary information (S1) to

**Disentangling mechanisms behind chronic lethality through toxicokinetic-toxicodynamic modelling**


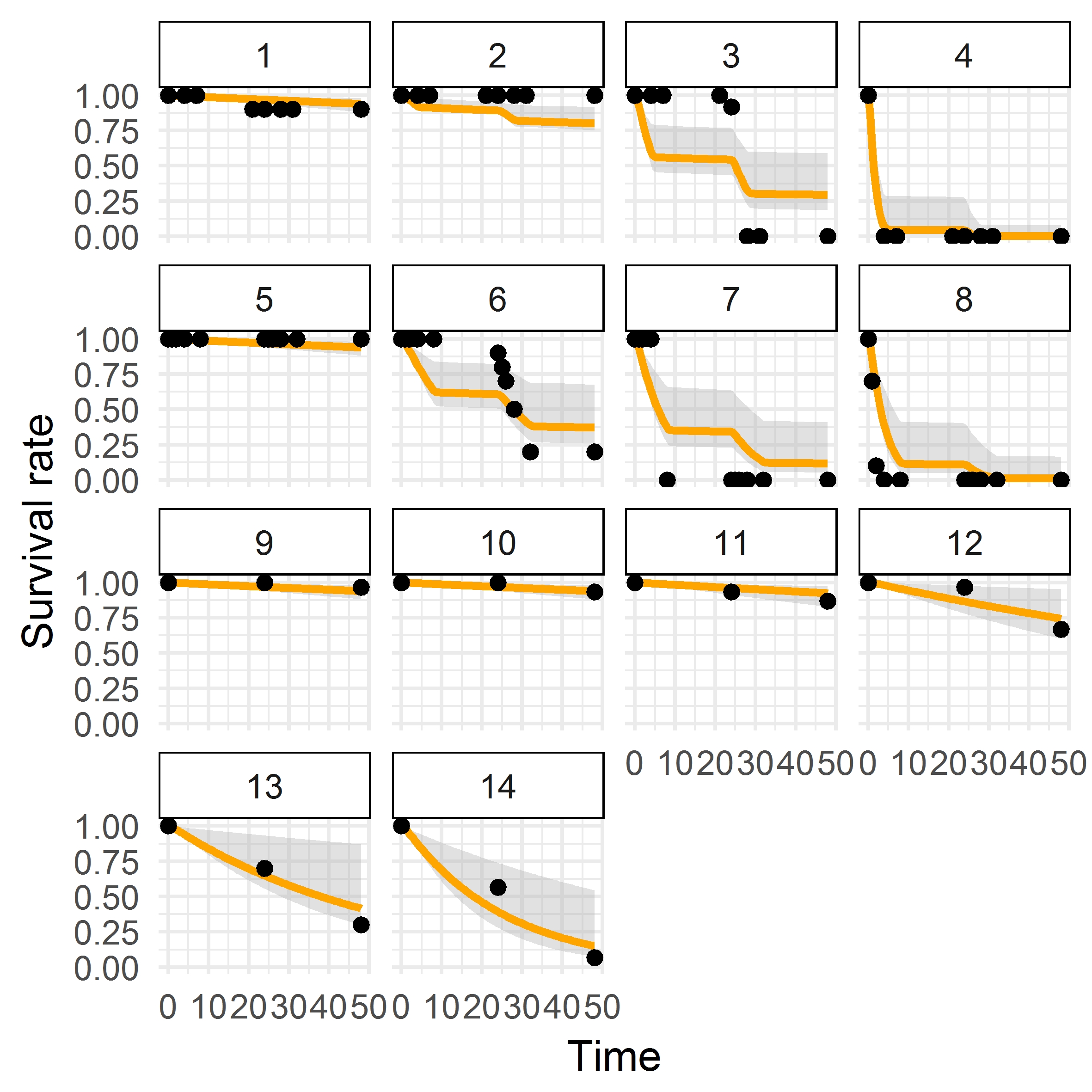


Figure S1-1: GUTS-SD calibration results for *Chironomus riparius* and **flupyradifurone** using time variable (1-8) and constant (9-14) exposures. For detailed scenarios, concentrations and survival data see Table S2-1 (in Supplementary Information S2). Unit of time is in hours.


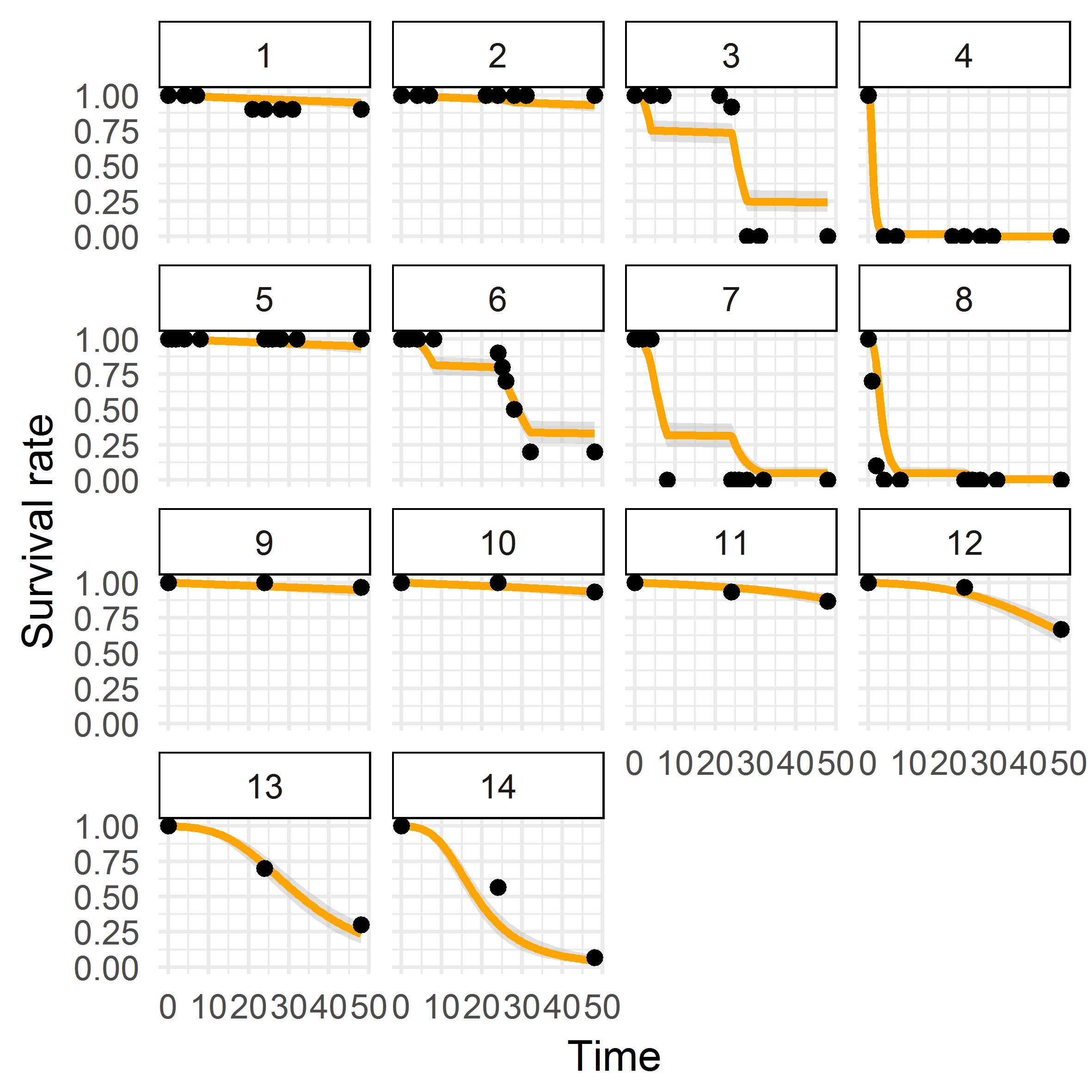


Figure S1-2: GUTS-IT calibration results for *C. riparius* and **flupyradifurone** using time variable (1-8) and constant (9-14) exposures. For detailed scenarios, concentrations and survival data see Table S2-1. Unit of time is in hours.

Table S1-1: GUTS-SD model evaluation criteria for **flupyradifurone**: posterior predictive check (PPC), normalized root mean square error (NRMSE), and survival predictive error (SPPE); see EFSA (2018) for details.

| criterion | value [%] |
| --- | --- |
| PPC | 91.5 |
| NRMSE | 43.1 |
| SPPE range | -25 - 20 |

Table S1-2: GUTS-SD parameter estimates for **flupyradifurone** (median and 95% confidence limits)

| parameters | median | lower | upper | unit |
| --- | --- | --- | --- | --- |
| kd | 2.59 | 0.857 | 11.2 | h^-1^ |
| hb | 0.00133 | 0.000440 | 0.00265 | h^-1^ |
| z | 0.0284 | 0.0122 | 0.0654 | mg L^-1^ |
| kk | 0.361 | 0.150 | 0.533 | L mg^-1^ h^-1^ |

kd: dominant rate constant; hb: background hazard rate; z: threshold for lethal effect; kk: killing rate

Table S1-3: GUTS-IT model evaluation criteria for **flupyradifurone**: posterior predictive check (PPC), normalized root mean square error (NRMSE), and survival predictive error (SPPE); see EFSA (2018) for details.

| criterion | value [%] |
| --- | --- |
| PPC | 96.8 |
| NRMSE | 25.5 |
| SPPE | -25 - 10 |

Table S1-4: GUTS-IT parameter estimates for **flupyradifurone** (median and 95% confidence limits)

| parameters | median | lower | upper | unit |
| --- | --- | --- | --- | --- |
| kd | 0.000764 | 0.00000904 | 0.00617 | h-1 |
| hb | 0.00113 | 0.000426 | 0.00223 | h-1 |
| alpha | 0.00189 | 0.0000226 | 0.0142 | mg L-1 |
| beta | 3.22 | 2.61 | 3.95 | - |

kd: dominant rate constant; hb: background hazard rate; alpha: median of threshold distribution; beta: slope of threshold distribution


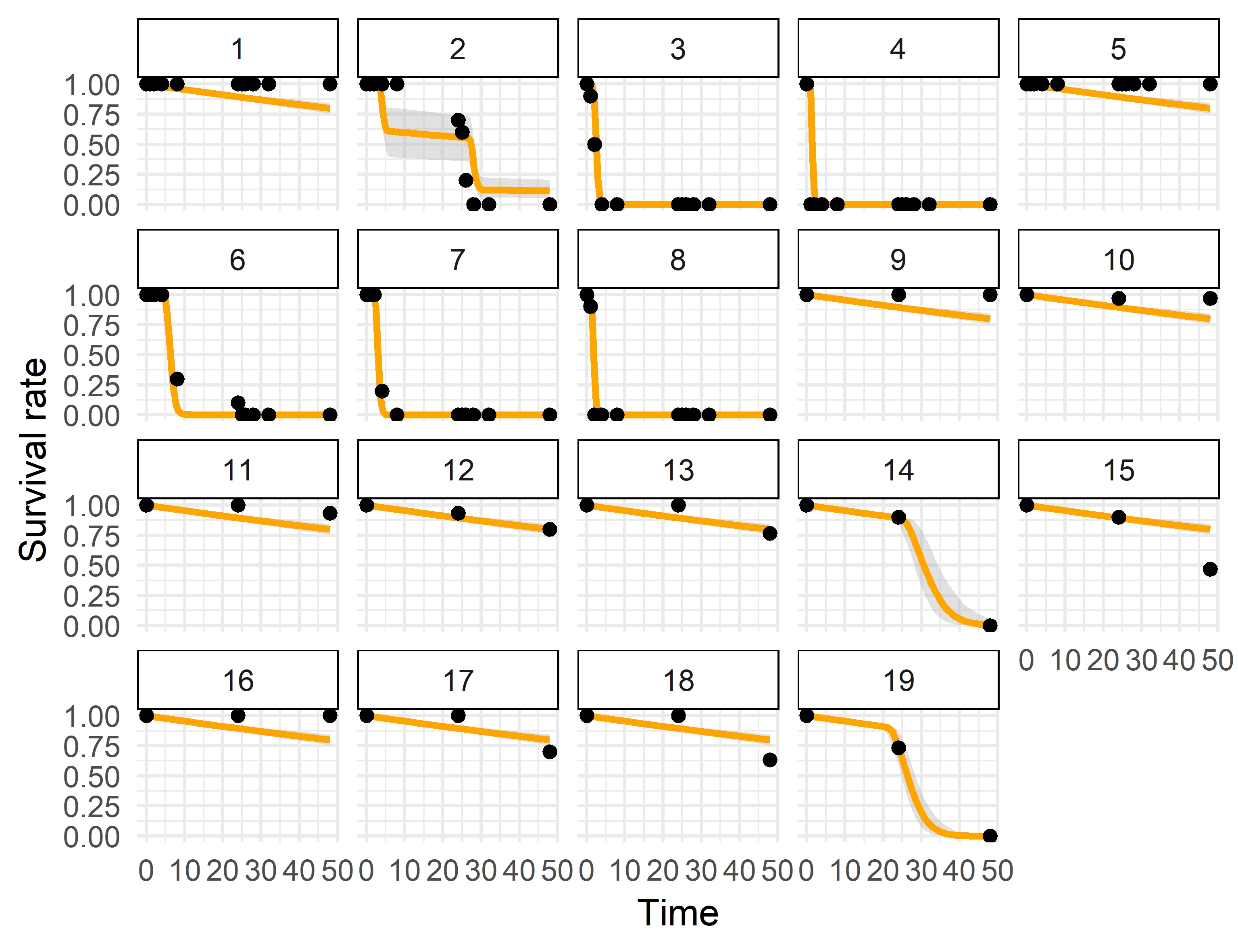


Figure S1-3: GUTS-SD calibration results for *C. riparius* and **imidacloprid** using time variable (1-8) and constant (9-19) exposures. For detailed scenarios, concentrations and survival data see Table S2-2. Unit of time is in hours.


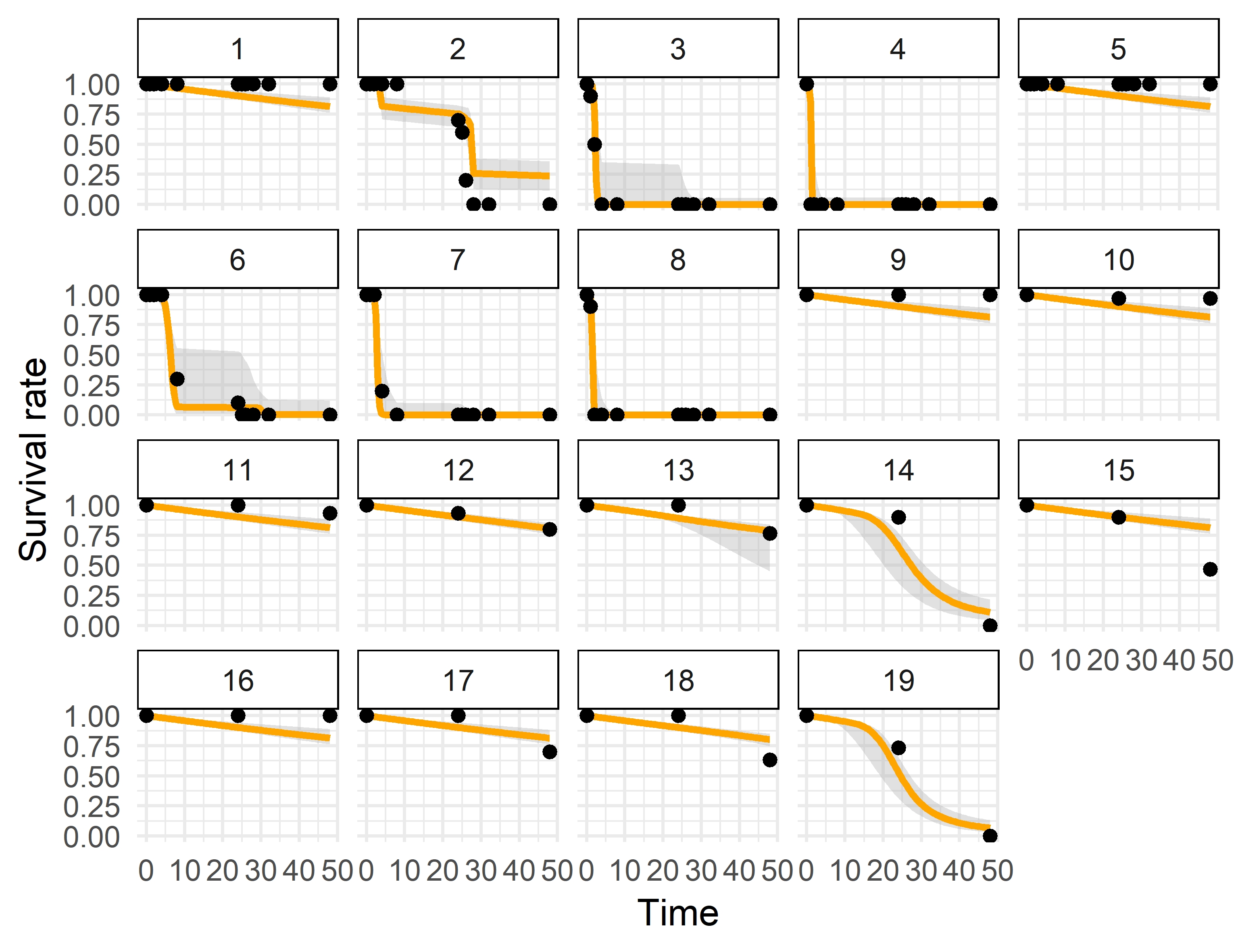


Figure S1-4: GUTS-IT calibration results for *C. riparius* and **imidacloprid** using time variable (1-8) and constant (9-19) exposures. For detailed scenarios, concentrations and survival data see Table S2-2. Unit of time is in hours.

Table S1-5: GUTS-SD model evaluation criteria for **imidacloprid**: posterior predictive check (PPC), normalized root mean square error (NRMSE), and survival predictive error (SPPE); see EFSA (2018) for details.

| criterion | value [%] |
| --- | --- |
| PPC | 93.4 |
| NRMSE | 35.5 |
| SPPE range | -33.3 - 20 |

Table S1-6: GUTS-SD parameter estimates for **imidacloprid** (median and 95% confidence limits)

| parameters | median | lower | upper | unit |
| --- | --- | --- | --- | --- |
| kd | 0.0914 | 0.0821 | 0.108 | h^-1^ |
| hb | 0.00469 | 0.00364 | 0.00590 | h^-1^ |
| z | 0.0502 | 0.0481 | 0.0522 | mg L^-1^ |
| kk | 64.8 | 36.9 | 105 | L mg^-1^ h^-1^ |

kd: dominant rate constant; hb: background hazard rate; z: threshold for lethal effect; kk: killing rate

Table S1-7: GUTS-IT model evaluation criteria for **imidacloprid**: posterior predictive check (PPC), normalized root mean square error (NRMSE), and survival predictive error (SPPE); see EFSA (2018) for details.

| criterion | value [%] |
| --- | --- |
| PPC | 95.0 |
| NRMSE | 44.9 |
| SPPE | -36.7 - 16.7 |

Table S1-8: GUTS-IT parameter estimates for **imidacloprid** (median and 95% confidence limits)

| parameters | median | lower | upper | unit |
| --- | --- | --- | --- | --- |
| kd | 0.0635 | 0.00371 | 0.0731 | h-1 |
| hb | 0.00431 | 0.00246 | 0.00568 | h-1 |
| alpha | 0.0472 | 0.00441 | 0.0509 | mg L-1 |
| beta | 13.5 | 3.25 | 20.1 | - |

kd: dominant rate constant; hb: background hazard rate; alpha: median of threshold distribution; beta: slope of threshold distribution


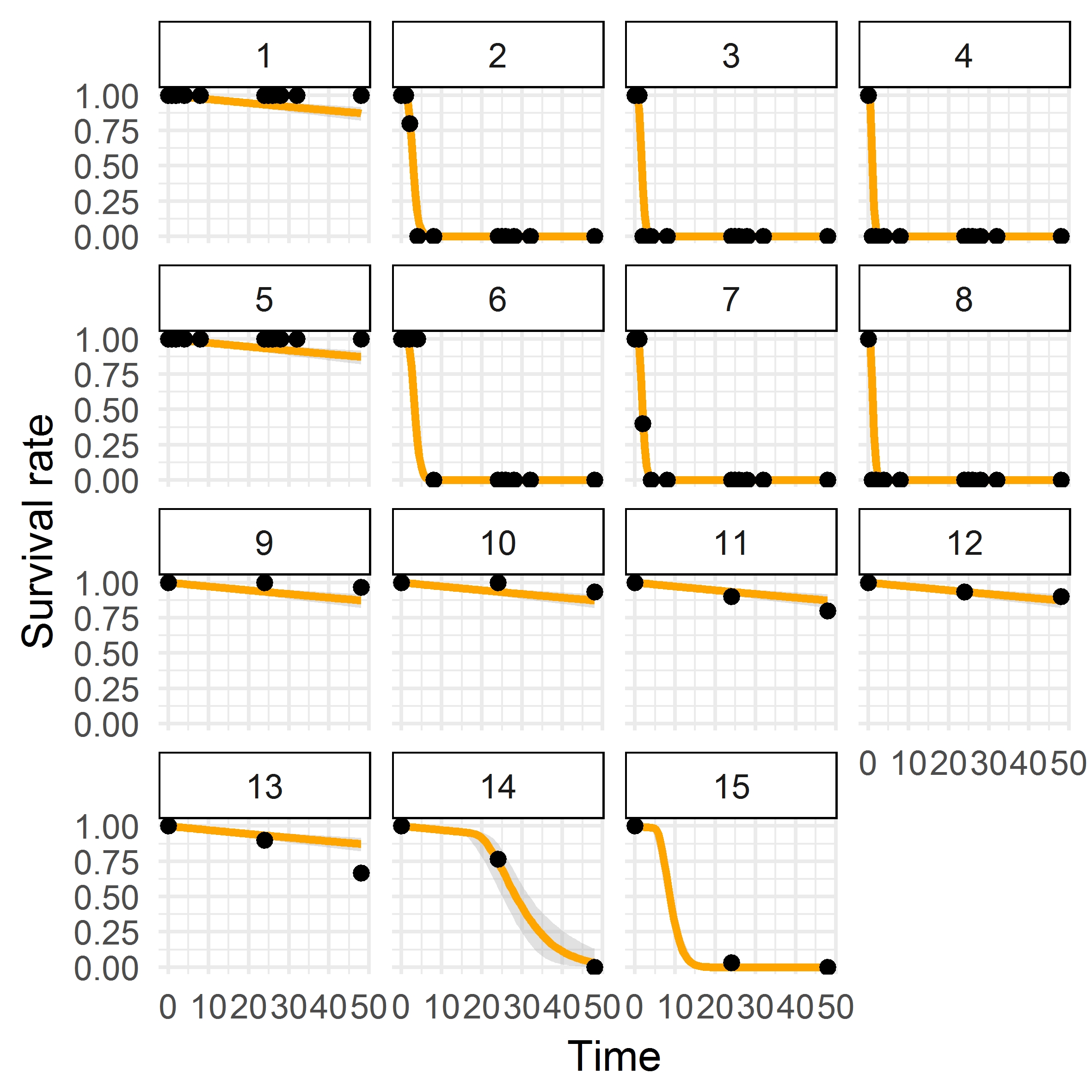


Figure S1-5: GUTS-SD calibration results for *C. riparius* and **thiacloprid** using time variable (1-8) and constant (9-15) exposures. For detailed scenarios, concentrations and survival data see Table S2-3. Unit of time is in hours.


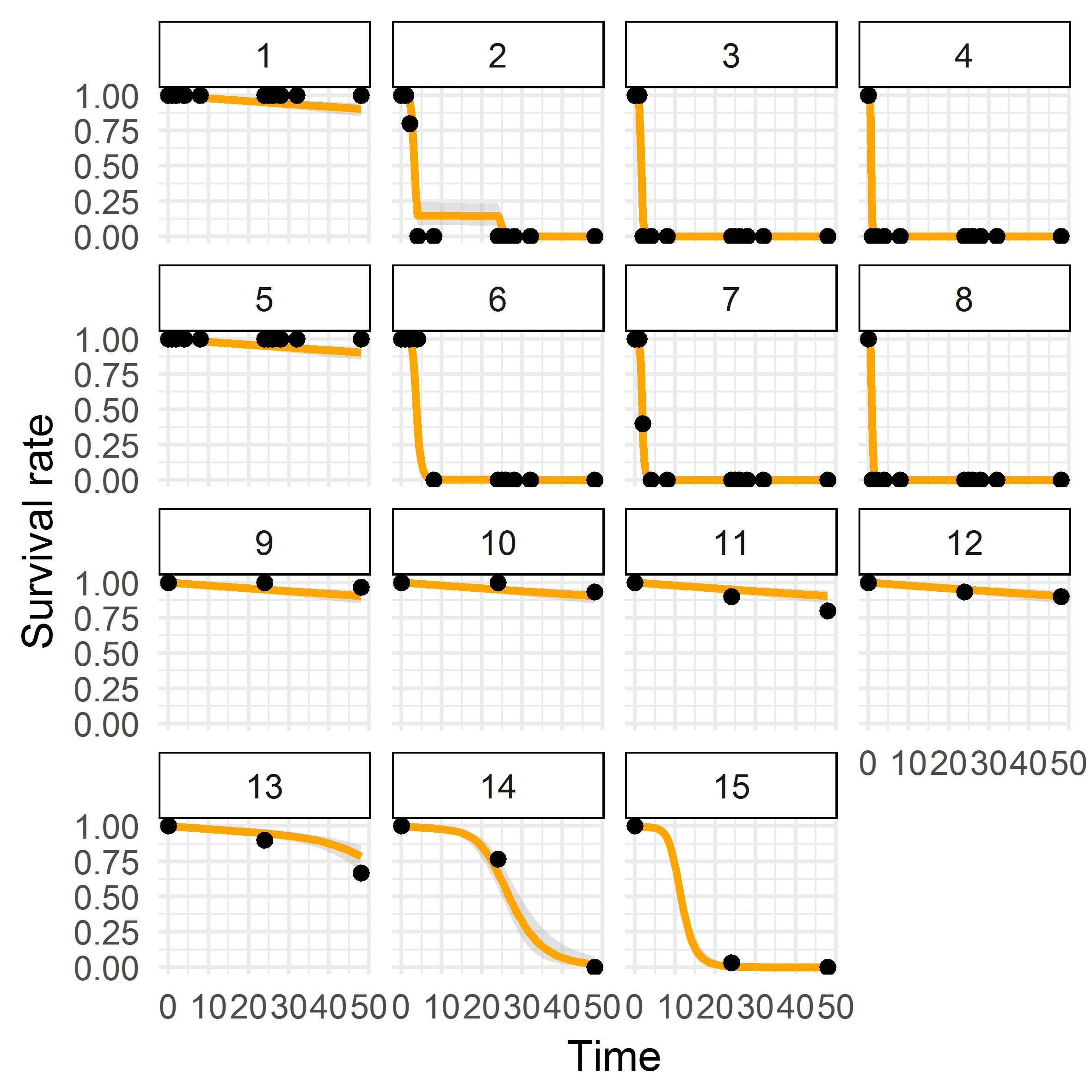


Figure S1-6: GUTS-IT calibration results for *C. riparius* and **thiacloprid** using time variable (1-8) and constant (9-15) exposures. For detailed scenarios, concentrations and survival data see Table S2-3. Unit of time is in hours.

Table S1-9: GUTS-SD model evaluation criteria for **thiacloprid**: posterior predictive check (PPC), normalized root mean square error (NRMSE), and survival predictive error (SPPE); see EFSA (2018) for details.

| criterion | value [%] |
| --- | --- |
| PPC | 95.4 |
| NRMSE | 21.8 |
| SPPE range | -6.7 - 10 |

Table S1-10: GUTS-SD parameter estimates for **thiacloprid** (median and 95% confidence limits)

| parameters | median | lower | upper | unit |
| --- | --- | --- | --- | --- |
| kd | 0.0881 | 0.0634 | 0.123 | h^-1^ |
| hb | 0.00281 | 0.00177 | 0.00416 | h^-1^ |
| z | 0.0179 | 0.0158 | 0.0195 | mg L^-1^ |
| kk | 35.6 | 21.4 | 59.2 | L mg^-1^ h^-1^ |

kd: dominant rate constant; hb: background hazard rate; z: threshold for lethal effect; kk: killing rate

Table S1-11: GUTS-IT model evaluation criteria for **thiacloprid**: posterior predictive check (PPC), normalized root mean square error (NRMSE), and survival predictive error (SPPE); see EFSA (2018) for details.

| criterion | value [%] |
| --- | --- |
| PPC | 98.2 |
| NRMSE | 13.4 |
| SPPE | -13.3 - 10 |

Table S1-12: GUTS-IT parameter estimates for **thiacloprid** (median and 95% confidence limits)

| parameters | median | lower | upper | unit |
| --- | --- | --- | --- | --- |
| kd | 0.00550 | 0.000897 | 0.0143 | h-1 |
| hb | 0.00211 | 0.00120 | 0.00338 | h-1 |
| alpha | 0.00314 | 0.000534 | 0.00775 | mg L-1 |
| beta | 7.26 | 5.56 | 9.71 | - |

kd: dominant rate constant; hb: background hazard rate; alpha: median of threshold distribution; beta: slope of threshold distribution

Table S1-13: Parameter values for the simulation of chronic effects. The chemical single first order dissipation rate (k) was employed in both GUTS and DEB simulations, while the threshold c_0_ and tolerance concentration c_T_ are DEB specific stress parameters. Note, that DEBtool, which was used for the estimation of DEB parameters, did not allow for the calculation of parameter uncertainty. The Mean Relative Error (MRE) and the Symmetric Mean Squared Error (SMSE) serve as measures for the goodness of model fit, see the DEB-wiki for information, at: http://www.debtheory.org/wiki/index.php?title=AmP_estimation_procedure#Goodness_of_fit_criterion

| Parameter/*criterion* | Flupyradifurone | Imidacloprid | Thiacloprid | Unit |
| --- | --- | --- | --- | --- |
| k | 0.0318 | 0.0779 | 0.136 | d-1 |
| c_0_ | 0.0196 | 0.0109 | 0.0169 | mg/L |
| c_T_ | 0.0530 | 0.0111 | 0.0228 | mg/L |
| *MRE* | 0.199 | 0.620 | 0.301 | - |
| *SMSE* | 0.329 | 1.16 | 0.657 | - |

c0: threshold concentration for sublethal effects, cT: tolerance concentration for sublethal effects

References

EFSA Panel on Plant Protection Products and their Residues (2018) Scientific Opinion on the state of the art of Toxicokinetic/Toxicodynamic (TKTD) effect models for regulatory risk assessment of pesticides for aquatic organisms. EFSA Journal 16 : doi:10.2903/j.efsa.2018.5377

List of underlying study reports

Detailed information of the material and methods as well as the data for flupyradifurone, imidacloprid and thiacloprid used in the current modelling exercise is available from the original study reports. The study reports can be requested by sending an email to: [cropscience-transparency@bayer.com](mailto:cropscience-transparency@bayer.com)

# *Flupyradifurone*

Bayer report 2016. Acute toxicity of flupyradifurone FS 480 G to larvae of Chironomus riparius in a 48 h static laboratory test system. Report No. M-545716-01-1

Bayer report 2017. Flupyradifurone – Acute toxicity to larvae of Chironomus riparius in a laboratory test under pulsed exposure conditions (non-GLP study). Two 4 hrs peaks. Report No. M-688850-01-1

Bayer report 2020. Flupyradifurone – Acute toxicity to larvae of Chironomus riparius in a laboratory test under pulsed exposure conditions (non-GLP study). Two 8 hrs peaks. Report No. M-688530-01-1

Bayer report 2011. Chironomus riparius 28-day chronic toxicity test with BYI 02960 (tech.) in a water-sediment system using spiked water. Report No. M-401792-01-2

Bayer report 2011. Chironomus riparius 28-day chronic toxicity test with BYI 02960 SL 200 G in a water-sediment system using spiked water. Report No. M-416145-01-1

# *Imidacloprid*

Bayer report 2016. Acute toxicity of imidacloprid WS 70 W to larvae of Chironomus riparius in a 48 h static laboratory test system. Report No. M-556343-01-1

Bayer report 2016. Acute toxicity of imidacloprid tech. (BCS-BB58951) to larvae of Chironomus riparius in a 48 h static laboratory test system. Report No. M-572014-01-1

Bayer report 2020. Imidacloprid – Acute toxicity to larvae of Chironomus riparius in a laboratory test under pulsed exposure conditions (two 4 hrs peaks) (non-GLP study). Report No. M-688529-01-1

Bayer report 2020. Imidacloprid – Acute toxicity to larvae of Chironomus riparius in a laboratory test under pulsed exposure conditions (two 8 hrs peaks) (non-GLP study). Report No. M-688648-01-1

Bayer report 2011. Chironomus riparius 28-day chronic toxicity test with BYI 02960 (tech.) in a water-sediment system using spiked water. Report No. M-366331-01-1

Bayer report 2004. Chironomus riparius 28-day chronic toxicity test with Imidacloprid SL 200, NMP-free in a water-sediment system using spiked water. Report No. M-091810-01-1

Bayer report 2004. Chironomus riparius 28-day chronic toxicity test with imidacloprid OD 200 in a water-sediment system using spiked water. Report No. M-092982-01-1

Bayer report 2003. Chironomus riparius 28-day chronic toxicity test with Imidacloprid WG 70 in a water-sediment system using spiked waterM-103625-01-1

*Thiacloprid*

Bayer report 2017. Acute toxicity of Thiacloprid (tech.) to larvae of Chironomus riparius in a 48 h static laboratory test system. Report No. M-491257-02-1

Bayer report 2020. Thiacloprid – Acute toxicity to larvae of Chironomus riparius in a laboratory test under pulsed exposure conditions (two 4 hrs peaks) (non-GLP study). Report No. M-688651-01-1

Bayer report 2020. Thiacloprid – Acute toxicity to larvae of Chironomus riparius in a laboratory test under pulsed exposure conditions (two 8 hrs peaks) (non-GLP study). Report No. M-688652-01-1

Bayer report 2003. Chironomus riparius 28-day chronic toxicity text with Thiacloprid 240 OD in a water-sediment system using spiked water. Report No. M-111299-01-1

Bayer report 2009. Chironomus riparius 28-day chronic toxicity test with thiacloprid FS 400 G in a water-sediment system using spiked water. Report No. M-361244-01-1
